# Influence of Fine and Coarse Waste Tyre Rubber Aggregates on the Workability, Mechanical Properties and Rubber–Paste Interface of Concrete: An SEM/EDX Study

**DOI:** 10.3390/ma19194060

**Published:** 2026-09-23

**Authors:** Marouan Bajbouji, Khalid Rkha Chaham, Saad Bensallam, Abdelmalek Goumih, Imane Mahboub

**Affiliations:** 1 Laboratoire Sciences de l’Eau, Biotechnologies Microbiennes et Durabilité des Ressources Naturelles, Département de Géologie, Faculté des Sciences Semlalia, Université Cadi Ayyad, Marrakech 40000, Morocco; rkha@uca.ac.ma (K.R.C.); a.goumih@uca.ac.ma (A.G.); imane.mahboub@uca.ac.ma (I.M.); 2 Department of Roadways and Transport (PCT), École Hassania des Travaux Publics (EHTP), Km 7, Route d’El Jadida, B.P. 8108, Casablanca 20250, Morocco; saad.bensallam@ehtp.ac.ma

**Keywords:** rubberised concrete, waste tyre recycling, interfacial transition zone, SEM/EDX, mechanical strength, workability, sustainable construction

## Abstract

**Highlights:**

- Coarse rubber decreases strength far more than fine rubber at equal volume replacement.
- Compressive strength fell from 41.3 to 7.5 MPa as replacement rose from 0 to 50%.
- Every rubber interface is portlandite-enriched versus bulk paste (Ca/Si 3.3–8.1 vs. 1.24).
- Strength loss has two causes: weakening of the ITZ and increased air voids at high coarse-rubber content.
- 10 vol. % fine rubber reduces compressive strength by less than 9%, viable for semi-structural concrete.
- Coarse rubber at 40–50 vol. % yields densities below the 2000 kg/m^3^ lightweight threshold.
- Portlandite-rich interfaces identify the target for rubber surface treatment.
- Particle size, not dosage alone, should govern mix selection for rubberised concrete.
- Supports graded valorisation of end-of-life tyres across construction applications.

**Abstract:**

Waste tyre rubber serves as a cost-effective aggregate substitute. The elemental-level interfacial mechanisms underlying the associated strength reduction remain insufficiently characterised. In this study, eleven concrete mixes were evaluated, with fine-rubber (2.5–5 mm) replacing river sand and coarse-rubber (5–20 mm) replacing crushed limestone, each at 10, 20, 30, 40 and 50% by volume. Cement content was maintained at 400 kg/m^3^ and the water-to-cement ratio at 0.45. Workability, hardened density, and 28-day compressive and flexural strengths were measured in accordance with Moroccan Standards (NM), which are aligned with the European Standards (EN). Scanning electron microscopy with energy-dispersive X-ray (SEM/EDX) microanalysis was conducted on six mixes to characterise the rubber–paste interfacial transition zone (ITZ). Compressive strength decreased by 8.5% with 10 vol. % fine rubber replacement and by 81.8% with 50 vol. % coarse rubber replacement, with coarse rubber consistently resulting in greater strength loss at all levels. Every qualifying paste-side field in the rubberised mixes returned a Ca/Si ratio above the reference bulk-paste value of 1.24, reaching 8.09, indicating portlandite accumulation at the hydrophobic rubber surface. Sulphur and zinc, introduced during vulcanisation, confirmed these regions as rubber rather than cement paste. The apparent debonding-zone width at the fracture surface increased from 10–25 µm to 100–200 µm and scaled with particle size, consistent with a flaw-size effect rather than with total interfacial area. Preliminary guidance on mix selection based on 28-day mechanical performance is proposed.

## 1. Introduction

End-of-life tyres (ELTs) have become a long-term, global environmental challenge. More than 1 billion tyres are discarded each year, and the figure continues to rise [1,2].

In regions with weak regulatory oversight, improper disposal practices such as open-air landfilling and incineration readily generate multiple hazards, including soil and groundwater pollution, habitat destruction, and pollutant emissions from tyre fires [2,3,4]. Two mainstream resource-recovery pathways exist. In the first, waste rubber is recycled and processed into construction materials. The design of recycled aggregate mixtures for this use is largely supported by predictive and statistical models [5], which can simultaneously reduce the demand for natural aggregates and alleviate the pressure on waste tyre disposal [6,7,8]. Preliminary investigations into the effects of replacing rubber particles on workability and mechanical properties have highlighted the potential for sustainable construction [9]. In the second, ELTs are applied in geotechnical engineering, where direct shear tests have identified an optimal rubber content of approximately 20% for sand–rubber mixtures [10,11]. The present study contributes to a regional tyre resource-recovery value chain serving both fields.

Rubber has a particle density of 1.1–1.2 Mg/m^3^ against 2.6–2.7 Mg/m^3^ for the natural aggregate [10,12], thereby lowering the density of the concrete and reducing its strength. Set against these losses, rubber improves concrete’s ductility, toughness, impact resistance, and energy absorption, making it suitable for applications such as road barriers, railway sleepers, playground surfaces, and acoustic panels [12,13]. The workability of rubberised concrete is jointly determined by the size, shape, and proportion of rubber [14,15]. Currently, the mainstream optimisation pathways for this material fall into three categories: supplementary cementitious materials [16]; fibre reinforcement of rubberised matrices [17,18]; and rubber surface pre-treatment [19,20].

The interfacial transition zone (ITZ) is the weakest region in concrete [21]. Natural aggregate is inherently more porous and less stiff than the bulk paste, a consequence of the wall effect and the accumulation of water against the aggregate surface. Around rubber, the zone is further weakened: the smooth, hydrophobic, non-porous surface suppresses the formation and adhesion of hydration products [22,23,24], producing a wider, more porous interface that serves as a pathway for crack initiation and growth. Similar interfacial weakening is reported for other low-density inclusions, for example, lightweight aggregate, where the porosity and chemistry of the rim govern strength [25]. Despite its central role, the rubber–paste ITZ is rarely characterised at the elemental level in studies that rely solely on macroscopic testing, leaving a gap between measured performance and the underlying mechanism [21,26].

Backscattered-electron imaging (BSE/QBSE) provides atomic-number contrast that allows phases and porosity to be distinguished, and energy-dispersive X-ray analysis (EDX) maps the elemental composition across the interface [27,28]. Used together, SEM and EDX can show how rubber content and particle size affect the morphology, width, and chemistry of the ITZ, thereby connecting the microstructure to macroscopic trends [29,30]. The present work combines macroscopic testing with SEM/EDX on six mixes, with four aims: to quantify workability, strength and density as functions of rubber content and type; to characterise the rubber–paste interface, including measurement of the apparent debonding-zone width at fracture surfaces; to assess porosity qualitatively; and to propose preliminary mix selection guidance based on mechanical performance.

## 2. Materials and Methods

### 2.1. Materials

The materials used in this experimental programme were sourced from local suppliers and selected to conform to the Moroccan Standards (NM), which are aligned with the European Standards (EN).

#### 2.1.1. Cement

Ordinary Portland cement of class CEM I 42.5 N, conforming to NM EN 197-1, served as the binder at 400 kg/m^3^ in each mix. With a water-to-cement ratio (w/c) of 0.45, this gave strength class C30/37, a standard general-purpose structural class.

The cement had a particle density of 3.15 Mg/m^3^. Setting began after 105 min and was complete after 180 min, and the standard mortar reached 46.7 MPa at 28 days. Utilising CEM I 42.5 N exclusively, without supplementary cementitious materials, enables the study to isolate the mechanical effects of rubber aggregates and avoid the confounding influence of secondary pozzolanic reactions [16].

#### 2.1.2. Fine Aggregate (Sand)

The fine aggregate was natural siliceous river sand of 0/4 mm grading from the Carrière GTR quarry on Oued Souss, a locally standard concrete sand selected for its regional availability and its established use in production concrete in the Marrakesh region. The sand-equivalent and methylene-blue values confirm a clay and fines content well within the limits for structural concrete. This matters because any variability in the sand would propagate directly into the comparison between mixes.

#### 2.1.3. Coarse Aggregate (Crushed Stone)

The coarse aggregate was crushed limestone of 5/20 mm grading from the same quarry, supplied in three sub-fractions of 4/10, 10/14 and 14/20 mm and recombined to a fixed grading in every mix. The 10/14 and 14/20 sub-fractions were characterised together as a combined 10/20 sample. Crushed limestone was selected as the reference coarse aggregate on two grounds: it is the material in general structural use in the region, and its angular, rough, hydrophilic surface represents the geometric and chemical opposite of the smooth, hydrophobic rubber that replaces it, rendering the interfacial contrast examined in Section 3.2 as sharp as the materials allow. Properties are given in Table 1, and all values satisfy the relevant specification limits. All eleven mixes drew on a single delivery from one quarry face, which removes aggregate source as a variable in the comparison.

#### 2.1.4. Tyre-Derived Aggregates (TDA)

The core experimental material of this study is rubber aggregate produced by mechanically grinding scrap tyres, with no chemical or physical surface pre-treatment applied. This reflects the most common low-cost production scenario and gives a baseline against which future surface treatments can be assessed [19,20]. Fine rubber (FR, 2.5–5 mm) replaced sand and coarse rubber (CR, 5–20 mm) replaced crushed stone. Both have a particle density of 1.15 Mg/m^3^ and negligible water absorption, and both were proportioned by volume as described in Section 2.2. The same granulate stream has previously been characterised at the L.3E. laboratory in sand–rubber mixtures for geotechnical use [10,11]. The smooth, hydrophobic rubber surface contrasts with the rough, hydrophilic surface of the natural aggregate, and the elastic modulus of rubber is three to four orders of magnitude below that of the mineral aggregate and the hardened paste [13,24]. These two contrasts govern the interfacial and mechanical behaviour examined in Section 3.1 and Section 3.2.

#### 2.1.5. Superplasticiser and Water

A polycarboxylate-based superplasticiser complying with NM EN 934-2 was used, dosed at 2.0–2.6 kg/m^3^. Water was fixed at 180 kg/m^3^, and the water-to-cement ratio was held at 0.45 for all mixes. The superplasticiser dose was raised in steps as rubber content increased to keep the fresh mixes placeable on the vibrating table without altering the water content. Slump was not equalised and was allowed to vary freely as a measured response. The consequences of the dose variation for the interpretation of the results are addressed in Section 3.1.5. Potable tap water complying with NM EN 1008 was used at 20 ± 2 °C.

### 2.2. Concrete Mix Design

Eleven mixes were prepared: a rubber-free reference mix (M0) and ten mixes in which either the fine aggregate was replaced by fine rubber (M1–M5) or the coarse aggregate by coarse rubber (M6–M10), at 10, 20, 30, 40 and 50% by volume (Table 2). Replacement was made on a volume basis: the particle density of rubber is about half that of the natural aggregate, and a mass substitution would alter the granular skeleton. The cement content (400 kg/m^3^) and water content (180 kg/m^3^) were identical in all mixes; only the replaced aggregate, the rubber, and the superplasticiser dosage varied (Table 2).

### 2.3. Mixing, Specimen Preparation, Curing and Testing

Each batch was prepared in a Diem-Werke DZ 120 V vertical-shaft forced-action pan mixer (196 L pan, 120 L batch capacity, 4 kW, 61 rpm; Diem-Werke GmbH, Hörbranz, Austria). The dry constituents, comprising cement, natural aggregates and the corresponding tyre-derived aggregate fraction, were mixed for 1 min to obtain a uniform distribution of the solid constituents. The predetermined quantity of mixing water was premixed with the required dosage of superplasticiser, and the resulting water–superplasticiser mixture was introduced into the mixer. Wet mixing continued for 3 min, giving a total mixing duration of 4 min. The same procedure was applied to every mixture, with only the superplasticiser dosage adjusted according to the proportions given in Table 2.

Immediately after mixing, the fresh concrete was discharged and its workability determined by the slump test (NM EN 12350-2). The concrete was placed into moulds and compacted on a vibrating table. Specimens were demoulded after 24 h and cured by water immersion at 20 ± 2 °C until testing at 28 days (NM EN 12390-2).

Compressive strength was determined at 28 days on cylinders measuring 150 × 300 mm, in accordance with NM EN 12390-3, at a loading rate of 0.6 MPa/s. Flexural strength was determined at the same age by three-point bending on prisms measuring 100 × 100 × 500 mm, according to NM EN 12390-5 at a loading rate of 0.05 MPa/s. Hardened density was determined under saturated surface-dry conditions (NM EN 12390-7). Three specimens were tested for each mixture and each property, with results reported as the mean ± one standard deviation. Any specimen deviating by more than 15% from the mean of its set was to be discarded and replaced by a re-tested specimen; no specimen met this condition in the present programme.

### 2.4. SEM/EDX Microstructural Characterisation

Fragments were cut with a diamond saw from the core of 28-day specimens. To preserve the native interfacial morphology, the fragments were not ground or polished; fresh fracture surfaces obtained by splitting were examined, following the general approach of ASTM C1723. Imaging and analysis were performed on a TESCAN VEGA3 LMU scanning electron microscope (TESCAN Orsay Holding, Brno, Czech Republic) fitted with an EDAX energy-dispersive X-ray detector (EDAX Inc., Mahwah, NJ, USA), operating in secondary-electron (SE) mode at an accelerating voltage of 10 kV, a live time of 30 s, an amplifier time of 1.92 µs, and a resolution of 129.4 eV. SE imaging shows topography rather than atomic-number contrast, and is used here for morphology only; quantitative phase and porosity segmentation would require BSE/QBSE imaging on polished sections [27,31]. For each mix, at least one bulk-paste area and one interfacial area were acquired, with additional areas on the rubber surface and the adjacent paste in M1, M3, M5, M6 and M10.

Apparent debonding-zone widths were measured directly from the calibrated micrographs using image-analysis software, with the scale set from the micrograph scale bar. For each rubber-bearing mix, the rubber–paste separation was measured perpendicular to the boundary, and the values are reported as a range. The quantity obtained on a fracture surface is the open separation exposed after splitting. It is not equivalent to the intrinsic ITZ thickness determined on polished sections. Fracture propagates preferentially along the weakest path and both exposes and enlarges the interfacial void, so the reported values constitute an upper bound on the physical interfacial thickness and are designated accordingly.

Polished-section measurements on sectioned rubberised composites report approximately 2 µm at powder-rubber margins and 8.8 µm at crumb-rubber edges [28], and a 2–9 µm range [32]. The values reported in Section 3.2 exceed those figures by roughly an order of magnitude, a difference consistent with fracture-path opening rather than with a genuinely thicker interfacial zone. These measurements are used to establish the ordering between mixes and are not interpreted as absolute interfacial thicknesses. Statistically robust, gradient-resolved profiling would require polished sections imaged in backscattered-electron mode [27,31], and verification against such imaging is identified in Section 3.2.10 as a requirement for further work.

Elemental quantification used the standardless eZAF Smart Quant routine (ZAF corrections), with results in weight per cent. Analysed fields were classified before interpretation according to a fixed rule. A field was assigned to the paste-side main sequence when it lay within approximately 20 µm of the rubber boundary on the paste side, returned a detectable silicon signal, and contained less than 10 wt. % carbon, the carbon limit excluding fields whose interaction volume demonstrably overlaps the rubber. Fields failing any criterion are retained and reported separately as secondary areas. A Ca/Si weight ratio in the range 0.8–2.0 indicates a load-bearing C–S–H gel, and a ratio above 2.0 indicates the accumulation of portlandite [30,33]. A sharp decrease in Ca/Si coinciding with a rise in carbon marks the transition toward the rubber surface. One or two fields qualified per mix, so Ca/Si is reported as a qualitative indicator of interfacial character rather than a quantitative dose–response variable.

### 2.5. Analytical Framework

Relative strength losses are expressed against the reference mix as Δ*f* = (*f*_0_ – *f*_i_)/*f*_0_, where *f*_0_ is the reference value and *f*_i_ the value for mix *i*. The sensitivity of the two strength measures is compared through the ratio

(1) *R* = Δ*f*_t_/Δ*f*_c_

and, equivalently, through the attenuation margin |Δ*f*_c_| − |Δ*f*_t_| expressed in percentage points, a percentage point being the arithmetic difference between two percentages.

Batch density is computed for each mix from the constituent masses of Table 2 on a zero-void assumption,

(2) *ρ*_batch_ = Σ_i_ *m*_i_

where *m*_i_ are the constituent masses per cubic metre, and is compared with the measured saturated surface-dry density *ρ*_SSD_ through the density deficit

(3) *D* = (*ρ*_batch_ − *ρ*_SSD_)/*ρ*_batch_ × 100

## 3. Results

### 3.1. Mechanical Properties

#### 3.1.1. Workability

Slump values for the eleven mixes are given in Table 3. The fine-rubber series rises above the reference value of 100 mm at 10 and 20 vol. % replacement, peaking at 114 mm, then falls to 55 mm at 50 vol. % replacement, a reduction of 45%. The initial increase can be linked to the rubber’s near-zero water absorption and smooth surface, which reduce interparticle friction and increase the amount of free water in the paste at a fixed water content of 180 kg/m^3^ [12,15,34]. The superplasticiser dose rose by 0.1 kg/m^3^ between M0 and M1, and the contribution of that increase to the observed slump gain requires bounding. M6 received 0.2 kg/m^3^ more than the reference mix, twice the increment applied to M1, and returned the reference slump of 100 mm. The admixture increment alone does not account for the 14 mm gain measured at M1, and the reduced interparticle friction of the smooth, non-absorbent fine rubber remains the governing contribution. The two influences are not separable in absolute terms within the present design. Above 20 vol. % replacement, the increasing proportion of light, deformable particles increases the mix’s viscous resistance, and particle friction becomes the dominant factor [35,36]. In the coarse-rubber series, the slump remained at the reference value at 10 vol. % replacement (M6, 100 mm) but declined steadily at higher replacement levels, reaching 85 mm at 20%, 70 mm at 30%, and 30 mm at 50% (M10, a 70% reduction). This decline is due to the interlocking of large, irregular granules as their volume fraction increases [14,37].

#### 3.1.2. Compressive Strength

Compressive strengths at 28 days and the corresponding relative reductions are given in Table 3 and plotted in Figure 1a. Both series decline monotonically with replacement level. The fine-rubber series decreases from 41.3 MPa in the reference mix to 13.9 MPa at 50% replacement, a loss of 66.3%, with the reduction modest at 10 vol. % replacement and steepening thereafter. The coarse-rubber series yields lower strength than the fine series at every replacement level, reaching 7.5 MPa at 50% replacement, a loss of 81.8%. The comparison at matched replacement is the informative one: 28.5 MPa for coarse rubber against 37.8 MPa for fine rubber at 10%, a difference of 9.3 MPa attributable to particle size at equal volume of rubber.

The greater reduction in strength observed for coarse rubber is attributed to the larger volume of weak, deformable material introduced by each particle, as well as to the wider and weaker interface surrounding the larger granules (see Section 3.2). From a fracture-mechanics perspective, the critical stress required for brittle fracture decreases with the square root of the characteristic flaw size. Thus, the debonded interface surrounding a large rubber granule can act as a more severe strength-limiting flaw than the smaller defects associated with fine-rubber particles, despite the larger total interfacial area per unit volume in the fine-rubber series [21,23,24,38]. Variability in compressive strength also increases with rubber content, with the coefficient of variation rising from 2.9% for the reference mix to 9–11% for M8–M10. This greater variability is consistent with the matrix heterogeneity observed in Section 3.2.

#### 3.1.3. Flexural Strength

After 28 days, the flexural strength (modulus of rupture, NM EN 12390-5) decreased as rubber content increased in both series (Figure 1b, Table 3). Larger coarse particles introduce macroscopic discontinuities that reduce the tensile resistance of the section, and the weak interface surrounding them supplies preferential sites for crack initiation [38,39]. The relative losses in the two strength measures are compared in Table 3 through the ratio R defined in Equation (1).

The equivalent margin in percentage points, |Δ*f*_c_| − |Δ*f*_t_|, is plotted in Figure 2 against a ±2 pp band that represents the replicate scatter in Table 3. Flexural strength is retained better than compressive strength in M3, M4, M5, M8 and M10, where the margin reaches 5.9 to 12.0 pp, and R falls to 0.79–0.93. The two measures are not separable in M2, M6, M7 and M9, whose margins of −0.5 to 1.5 pp lie inside the band. M1 reverses the effect, flexural strength falling by 11.1% against a compressive loss of 8.5%, a margin of −2.6 pp. The attenuation is reported as a tendency confined to intermediate and high replacement rather than as a general property of the series.

Two mechanisms of opposite sign act on R, accounting for its inconsistency. Compressive failure of concrete proceeds by lateral tensile splitting and requires a continuous stiff skeleton to transfer load. The elastic modulus of tyre rubber lies three to four orders of magnitude below that of the hardened paste, so each granule behaves mechanically as a cavity. For a spherical cavity in an elastic matrix, the Goodier solution gives the maximum tangential tensile stress at the inclusion surface as

(4) *σ*_θ,max_ = [(27 − 15*ν*)/2(7 − 5*ν*)] *σ*_app_

where ν is the Poisson ratio of the matrix and σapp the remotely applied compressive stress. For ν = 0.2, the factor reduces to 2.0, so every granule seeds a transverse tensile field of roughly twice the applied stress. Compressive strength decreases with the volumetric density of such inclusions and with the progressive loss of load path through the aggregate framework.

Flexural failure is governed instead by the single most severe flaw within the tensile zone. Following the Griffith criterion in the Irwin form,

(5) *f*_t_ = *K*_IC_/*Y*(π*a*_max_)^1/2^ = (*E*′*G*_F_)^1/2^/*Y*(π*a*_max_)^1/2^

where *K*_IC_ is the fracture toughness, *a*_max_ the governing flaw size, *Y* a geometry factor, *E*′ the effective elastic modulus and *G*_F_ the fracture energy. The population of defects that drives compressive loss is not the quantity that sets flexural strength; a single flaw of size *a*_max_ does.

Equation (5) also contains the compensating term. Rubber lowers E′ and enlarges a_max_, both of which reduce flexural strength, yet its low stiffness contributes crack bridging, crack deflection, and crack-tip blunting, which raise G_F_. Compression offers no equivalent term in Equation (4), where the concentration factor is independent of inclusion stiffness once the cavity limit is reached. The attenuation observed at intermediate replacement follows from this asymmetry. The compensation is bounded by flaw size: coarse granules enlarge a_max_, and the inverse-square-root scaling of Equation (5) removes in flexural strength what the rise in G_F_ returns. R consequently approaches unity throughout the coarse series while attenuation persists in the fine series, and the same flaw-size argument accounts for the greater compressive penalty of coarse rubber discussed in Section 3.1.2.

#### 3.1.4. Hardened Density

The 28-day hardened density (saturated surface-dry) decreased from 2327 kg/m^3^ for the reference mix to 2140 kg/m^3^ with 50% fine rubber, representing an 8.0% reduction, and to 1926 kg/m^3^ with 50% coarse rubber, corresponding to a 17.2% reduction (Table 3, Figure 3a). This decrease is primarily due to the replacement of dense mineral aggregate (particle density 2.63–2.65 Mg/m^3^) by lighter rubber (1.15 Mg/m^3^). Each unit volume of rubber replacement removes approximately 56% of the mass of the aggregate it replaces [11,40].

The reduction is non-linear. M6 to M8 decrease gradually, while a drop of 189 kg/m^3^ separates M8 from M9. The change in constituent mass between the two mixes accounts for only 64.7 kg/m^3^ of that figure, leaving 124 kg/m^3^ unexplained by the exchange of natural aggregate for rubber. Placing this residual on a common basis through the density deficit D of Equation (3), D lies between 0.2% and 2.0% for M0 through M8, with no trend across that range, and rises to 6.3% and 5.6% for M9 and M10. The fine-rubber series shows no comparable step at any replacement level, so the discontinuity is confined to the coarse series at high dosage.

The saturated surface-dry condition directly affects how this deficit may be interpreted. Connected pores are water-filled at the time of weighing and contribute only the difference between water and solid density, so open porosity is substantially masked in the measurement. Entrapped voids isolated from the pore network remain air-filled and register in full. Accordingly, the deficit reported here is a lower bound on the total void content. Compaction failure at high coarse-rubber dosage, arising from the loss of a continuous stiff skeleton and the resulting difficulty of consolidating the fresh mix, is the interpretation most consistent with a step change confined to M9 and M10 and absent from the fine series [36,41].

This interpretation is an inference from the density record and not a measurement of porosity. Air content was not determined on the fresh mixes, and water absorption and open porosity were not measured on the hardened specimens. Direct confirmation requires the porosity testing identified in Section 3.2.10.

According to EN 206, M9 and M10 fall below the 2000 kg/m^3^ threshold and qualify as lightweight concrete, whereas M5 lies above it. The threshold in EN 206 is defined on oven-dry density, while the values reported here are saturated surface-dry and exceed the corresponding oven-dry figures; the classification of M9 and M10 is conservative on that basis, and oven-dry determination would be required to confirm the standing of the intermediate mixes. The classification of M9 and M10 arises in part from entrapped void rather than from substitution alone, and any dead-load benefit claimed on this basis requires confirmation that the void content is acceptable for the intended application.

#### 3.1.5. Superplasticiser Variation and Its Bearing on the Comparisons

The superplasticiser dose was not held constant across the series, rising from 2.0 kg/m^3^ in the reference mix to 2.6 kg/m^3^ at 50% coarse rubber. Strict single-variable control was not achieved, so the measured differences cannot be attributed solely to the rubber. Three considerations bound the influence of this variation. The total range corresponds to 0.50–0.65% of the cement mass, a difference of 0.6 kg/m^3^ across eleven mixes. At a fixed water content, the superplasticiser disperses the binder without altering hydration stoichiometry, and its effect on 28-day strength is neutral to marginally beneficial, so the higher doses accompany the mixes that lost the most strength, and the reported reductions represent conservative estimates.

The comparison between the two fractions of rubber is free of this confound. Four pairs of mixes received identical superplasticizer doses: M2 with M6 at 2.2 kg/m^3^, M3 with M7 at 2.3 kg/m^3^, M4 with M8 at 2.4 kg/m^3^, and M5 with M9 at 2.5 kg/m^3^. In each pair, the coarse-rubber mix contains 10 vol. % less rubber yet yields the lower compressive strength by 3.0, 0.6, 1.6 and 1.5 MPa, respectively (Figure 4). The particle-size effect reported in this study is established by these dosage-matched comparisons and does not depend on the series trend. A dedicated series at a fixed superplasticiser dose would be required to isolate the absolute magnitude of the rubber penalty within each series, and this is identified as further work.

### 3.2. SEM/EDX Microstructural Characterisation: Interface and Porosity

#### 3.2.1. Analytical Approach and EDX Data Summary

SEM and EDX analyses were conducted on M0, M1, M3, M5, M6, and M10 to investigate the bulk paste, morphology, composition, and apparent debonding-zone width at the rubber–paste boundary, and to qualitatively assess porosity. The elemental signals of carbon, oxygen, aluminium, silicon, and calcium were used to distinguish the principal constituents. Hydrates are identified as calcium- and silicon-rich; rubber as carbon-rich with a sulphur–zinc signature from vulcanisation; aggregate fines as silicon- and aluminium-rich; and pore space by a reduction in the overall signal. Table 4 presents the paste-side main sequence of fields that satisfy the criterion stated in Section 2.4; Table 5 reports the secondary areas excluded from that sequence; and Table 6 provides the measured apparent debonding-zone widths. Standardless quantification limits the interpretation to trends rather than absolute compositions [27,31,42].

**Table 4:** Paste-side main sequence: EDX composition (wt. %) and Ca/Si ratio for fields satisfying the Section 2.4 criterion.

Mix	Repl. Vol. %	Field	C	O	Al	Si	Ca	Ca/Si
M0	0%	Bulk paste (reference)	n.d.	41.87	6.81	22.87	28.46	1.24
M1	10% FR	Paste-side	8.21	41.08	5.30	10.53	34.88	3.31
M5	50% FR	Paste-side	5.97	41.06	3.94	18.35	27.90	1.52
M5	50% FR	Paste-side	n.d.	35.19	2.37	4.85	52.28	10.78
M6	10% CR	Paste-side	4.58	36.59	2.38	6.21	50.24	8.09
M6	10% CR	Paste-side	5.00	44.61	1.67	5.67	42.73	7.54
M10	50% CR	Paste-side	5.46	46.19	3.71	9.52	35.13	3.69

M3 contributes no qualifying field: its interfacial area contains 26.87 wt. % carbon, exceeding the 10 wt. % limit, and its second area is a bulk-paste field rather than an interface. Excluded fields are listed in Table 5.

**Table 5:** Secondary areas excluded from the paste-side main sequence, reported for completeness. The M1 rubber surface additionally returned S 2.67 wt. % and Zn 1.02 wt. %; an Fe-K signal of 2.14 wt. % was detected in one M5 area.

Mix	Field	Exclusion Ground	C	Si	Ca	Ca/Si
M1	Rubber surface	C 60.60 wt. %; no Si	60.60	n.d.	16.82	—
M3	Boundary-overlapping	C 26.87 wt. % > 10 wt. %	26.87	4.84	22.92	4.74
M3	Bulk paste	Not an interfacial field	6.51	7.51	36.85	4.91
M10	Rubber surface	C 52.93 wt. %	52.93	3.24	14.01	4.32

**Table 6:** Apparent debonding-zone width measured on the fracture-surface micrographs.

Mix	Replacement Vol. %	Apparent Debonding-Zone Width (µm)
M0	0% (ref.)	–
M1	10% FR	10–25
M3	30% FR	65–70
M5	50% FR	85–105
M6	10% CR	45–80
M10	50% CR	100–200

#### 3.2.2. M0 (Reference) Baseline Microstructure

The reference paste exhibits a dense, broadly homogeneous structure, typical of a well-hydrated CEM I paste after 28 days. The bulk composition reveals a Ca/Si ratio of 1.24. The relatively high silicon content suggests the incorporation of fine sand in the sample. In the analysed field, the absence of carbon confirms the absence of rubber, while the detection of aluminium is consistent with the presence of aluminate hydrates and feldspathic fines. This Ca/Si ratio is used as the bulk-paste reference to interpret interfaces in rubber-containing mixtures. A substantially higher ratio at a rubber–paste boundary indicates the accumulation of calcium hydroxide (CH) rather than calcium silicate hydrate (C–S–H), which is linked to weaker interfacial bonding (Figure 5a) [21,43].

#### 3.2.3. M1 (10 Vol. % FR) Incipient Interfacial Separation

The paste-side field returned a Ca/Si ratio of 3.31, substantially above the M0 bulk value (Table 4). This elevation indicates a thin portlandite-bearing layer at the interface, consistent with the wall effect and with water enrichment adjacent to a hydrophobic surface. The rubber surface was dominated by carbon and contained the sulphur and zinc characteristic of vulcanised tyre material (Table 5); the calcium detected there indicates partial, limited deposition of hydration products. The apparent debonding-zone width ranged from 10 to 25 µm, the narrowest among the rubber mixes (Table 6, Figure 5b). The narrow, only moderately enriched interface at 10% fine rubber corresponds to the minimal strength reduction measured in this mix [22,24].

#### 3.2.4. M3 (30 Vol. % FR) Widening Separation and Porosity Increase

A mixed interfacial area yielded a Ca/Si ratio of 4.74 at a carbon content of 26.87 wt. % (Table 5). The elevated carbon places this region at or across the rubber boundary, so the field is reported as a secondary area rather than in the paste-side main sequence. Traces of Na, Mg and K are attributed to cement alkalis or mineral inclusions. A second, nominally bulk-paste area yielded Ca/Si = 4.91, best interpreted as a sampling effect of the fracture surface rather than a change in paste stoichiometry: with 30% of the sand replaced, less sand is present in any given field, raising the likelihood that an area selected as paste intersects a portlandite-rich interfacial region. The intrinsic C–S–H chemistry remains set by the cement composition and the constant water-to-cement ratio. The apparent debonding-zone width increased to 65–70 µm, and the micrographs show increasing microporosity, with discrete pore clusters forming on the rubber surfaces, consistent with the 39.5% strength loss at this level (Figure 5c) [21,29].

#### 3.2.5. M5 (50 Vol. % FR) Advanced Interfacial Degradation

At 50 vol. % fine rubber, the matrix is markedly disrupted, and the two qualifying paste-side fields differ by a factor of seven (Table 4). The silicon-rich field is best interpreted as a well-polymerised C–S–H or residual fine-sand domain, captured in place of a portlandite shell, and the calcium-rich field as the opposite extreme. Three explanations are possible for a spread of this magnitude: genuine matrix heterogeneity at 50% replacement, fracture-surface topography with associated X-ray shadowing, and sampling bias arising from a limited number of fields. The present data do not distinguish among them. One observation bears on the question. The two M6 fields, acquired under identical conditions and the same selection criterion, agree to within 7%, whereas the M5 fields differ by a factor of seven; topographic and sampling effects apply equally to both specimens. On that basis, the heterogeneity reading is offered as a preliminary observation requiring confirmation by elemental area mapping or by an increased field count on polished sections, and it is not treated as an established result. The apparent debonding-zone width ranged from 85 to 105 µm (Figure 5d). These observations are consistent with the 66.3% reduction in compressive strength and the 59.3% reduction in flexural strength measured for this mix. The Fe-K signal (2.14 wt. %) detected in one area may indicate residual steel-cord fines from tyre processing.

#### 3.2.6. M6 (10 Vol. % CR): Coarse-Rubber Interface, Geometry Dominates

M6 carries coarse rubber at the same 10 vol. % replacement level as M1, yet yields 28.5 MPa compared to 37.8 MPa. The microstructure accounts for the difference. Its two interfacial fields yield the highest paste-side Ca/Si values in the study (Table 4), and a trace sulphur peak confirms the presence of rubber within the interaction volume. The apparent debonding-zone width of 45–80 µm is approximately three times that of M1 at the same replacement level. The explanation is geometric and centres on flaw size rather than interfacial area: coarse granules introduce larger compliant inclusions, each surrounded by a wider and more continuous interfacial defect, and their angular edges concentrate stress, so the debonded zone around a large particle acts as a more severe strength-limiting flaw than the fine-scale defects around small particles. Fine rubber presents a greater total interfacial area per unit volume, yet the damage at each interface is more severe for coarse particles. This size-dependent ordering is consistent with observations on sectioned rubberised composites (Figure 5e) [21,32].

#### 3.2.7. M10 (50 Vol. % CR) Severely Degraded Matrix

M10 carries the highest coarse-rubber content, 50 vol. %, and provides a second observation at that replacement level. The paste-side area of M10 yields a Ca/Si ratio of 3.69, which is substantially lower than the 8.09 measured at 10% coarse rubber. If the paste-side ratio were a monotonic function of rubber content, M10 would exhibit the highest value. The depressed value at the highest replacement level repeats the pattern seen in M5, where the single qualifying field also failed to return the highest ratio in its series. The consistent reading across the two 50% mixes is that a single field ceases to be representative of interfacial chemistry once replacement is high, whether the underlying cause is matrix heterogeneity or sampling of a non-representative domain. Both interpretations carry the same operational consequence for the present dataset, and neither is resolved by it. The rubber surface analysis aligns with this finding: the coarse rubber surface contains 14.01 wt. % calcium, compared with 16.82 wt. % on the M1 rubber, indicating that less hydration product is deposited on the coarse granules. The apparent debonding-zone width reached 100–200 µm, the widest in the study. Scanning electron microscopy shows a friable, highly porous material with microcracking visible around the particles, consistent with the observed 81.8% compressive and 75.9% flexural losses (Figure 5f) [23,24].

#### 3.2.8. Interfacial Chemistry and Widths Across the Series

In all rubber mixtures, the paste-side Ca/Si ratio exceeds the M0 bulk value, indicating enrichment of portlandite at the hydrophobic rubber surface (Figure 6a). This enrichment positions all interfaces within the poorly bonded range, as defined by reported bond thresholds of 1.15–1.62 [43] and 1.06–1.91 [32]. As the replacement level increases, the variability of individual readings also rises, reflecting the limited EDX field relative to the scale of heterogeneity. At 10% replacement, coarse rubber shows greater interfacial enrichment than fine rubber, and at 50% replacement both series return values below their series maxima (Table 4). No dose–response relationship is claimed for Ca/Si against replacement level; the non-monotonic behaviour at 50% replacement is discussed in Section 3.2.5 and Section 3.2.7 and bounded in Section 3.2.10. The measured apparent debonding-zone widths offer an independent and monotonic contrast to the scattered chemical data (Table 6, Figure 7). Width increases consistently with rubber content within each series, and at equivalent replacement the coarse-rubber value is approximately three times the fine-rubber value at 10% and about twice at 50 vol. %. The increasing variability at high replacement parallels the Ca/Si scatter. The comparison establishes the ordering between mixes and not the absolute scale, for the reasons set out in Section 2.4.

Two further observations support the reading set out above. Paste-side carbon rises from M1 to M3 (Table 4 and Table 5; Figure 6b) as the interface widens and a greater proportion of rubber enters the analysed volume, giving an index of interfacial growth independent of the Ca/Si measurement. The sulphur–zinc fingerprint on the M1 rubber surface, together with the trace sulphur at the M6 interface, associates the carbon-rich, poorly bonded phase with tyre material [30].

#### 3.2.9. Qualitative Assessment of Porosity

Porosity was evaluated qualitatively using secondary electron (SE) imaging of fracture surfaces. The micrographs indicate a significant increase in microporosity with increasing rubber content, with voids primarily concentrated at the ITZ [40,44].

This microstructural evolution progresses from isolated, discrete pores at low replacement levels to a network of clustered, interconnected voids in the M5 and M10 mixes. This is attributed to the hydrophobic, non-polar characteristics of shredded tyres, which repel water and facilitate air entrainment on the rough, irregular rubber surfaces during mixing [36]. Secondary-electron imaging records topography rather than atomic-number contrast, so this assessment is qualitative throughout; no pore fraction, pore-size distribution or connectivity is reported.

#### 3.2.10. Limitations of the SEM/EDX Analysis

The microstructural evidence in this study carries four constraints that bound the conclusions drawn from it. Specimens were examined as fresh fracture surfaces rather than polished sections, a choice made to preserve the native interfacial morphology. Fracture surfaces expose the weakest path through the material and open the rubber–paste boundary, so the widths reported in Table 6 are apparent debonding-zone widths and constitute upper bounds rather than measurements of intrinsic ITZ thickness. Their value lies in the ordering they establish between mixes.

Imaging in secondary-electron mode records topography and not atomic-number contrast. Phase identification and porosity segmentation both require backscattered-electron or quantitative backscattered-electron imaging on polished sections, and no such imaging was performed. The porosity assessment in Section 3.2.9 is accordingly qualitative throughout. Elemental quantification was standardless, which introduces well-documented uncertainty for light elements [45], and one to two fields qualified as main-sequence in each mix. Ca/Si is reported on that basis as a qualitative indicator distinguishing portlandite-enriched interfaces from C–S–H-dominated paste. No dose–response relationship is claimed for Ca/Si against replacement level, and the non-monotonic values at 50% replacement are interpreted in Section 3.2.5 and Section 3.2.7 as preliminary observations.

Water absorption, open porosity and transport testing were outside the scope of this study. Confirmation of the void mechanism inferred from the density record in Section 3.1.4 requires direct porosity measurement. Nuclear magnetic resonance relaxometry has been applied to resolve pore-structure evolution in cemented composites [46], and mercury intrusion porosimetry combined with quantitative backscattered-electron segmentation would supply the pore-size distribution and area fractions absent here. These measurements, together with backscattered-electron verification of the intrinsic interfacial thickness, are identified as the priority for further work.

### 3.3. Preliminary Mix Selection Based on 28 Days Mechanical Performance

The guidance below is derived from 28-day compressive strength, flexural strength and hardened density, together with the interfacial observations of Section 3.2. No durability testing was performed in this study. Water absorption, chloride and carbonation resistance, freeze–thaw durability and permeability were not measured, and the interfacial porosity documented in Section 3.2.9 gives specific reason to expect transport properties inferior to those of the reference mix. The guidance constitutes a preliminary ranking on mechanical grounds and does not constitute a specification. No mix in this study should be placed in service, and none should be assigned to any exposure class under EN 206, before the durability properties governing that exposure have been determined experimentally. The ranking is set out in Table 7. M1 is the most promising semi-structural mix on mechanical grounds, and its narrow, only moderately enriched interface is consistent with effective load transfer. M6 meets the C20/25 strength threshold but carries the highest measured interfacial enrichment and a wide debonding gap, so its mechanical ranking overstates its likely service performance; the entry is flagged accordingly. All entries are subject to the durability verification stated above.

## 4. Discussion

### 4.1. Mechanical Degradation in the Context of Published Strength Envelopes

The strength reductions observed in this study fall within the range reported for rubberised concrete over the past thirty years. Khaloo et al. [35] reported significant compressive strength losses at high tyre-rubber volume replacement, accompanied by a notable increase in ultimate strain. Ganjian et al. [12] observed a reduction of approximately one-third of the reference strength at their highest chipped-rubber dosage, which aligns with the 31.0% reduction observed for M6. Huang et al. [24] identified both substantial strength loss and increased toughness in chipped-rubber concrete. The fine-rubber series demonstrates less pronounced strength reduction at lower dosages, and M1 is positioned at the favourable end of the range reported in recent syntheses for 10% fine-rubber replacement [1,36,40]. The recommendation by Mujalli et al. [40] to limit crumb-rubber content to 10–20% for code-compliant strength classes is consistent with the placement of M1.

Particle size serves as a more definitive diagnostic criterion. Su et al. [48] demonstrated that, for singly sized rubber, smaller particles result in reduced strength loss at equivalent dosages. AlMousawi et al. [44] attributed the inferior performance of rubber chips compared with crumb and powder to the presence of gaps and microcracks around larger particles. Du et al. [47] identified fine-rubber particles near 0.5 mm as the least damaging size within a 0.1–20 mm test range. The observed penalty associated with coarse rubber (−31.0% at M6 compared to −8.5% at M1) aligns with this consensus and is supported by the microstructural analysis in Section 3.2. This is the flaw-size scaling of Equation (5) [21,38,49].

Flexure reduces the magnitude of losses without altering their sequence in part of the series. The 28-day flexural losses are smaller than the corresponding compressive losses in M3, M4, M5 and M8, indistinguishable in M6, M7 and M9, and larger in M1, as set out in Section 3.1.3. Wang et al. [39] derived tensile stress–strain relationships for rubberised concrete from flexural tests and similarly observed a moderation of the tensile-side penalty.

### 4.2. Comparison of the Rubber–Paste Interface with Independent ITZ Studies

The chemical characterisation of the interface is consistent with published EDX studies and permits direct comparison. Moustafa and ElGawady [43] measured a Ca/Si ratio of 0.37 at a natural-aggregate interface, compared with 1.15 and 1.62 at rubber interfaces, and identified ratios below approximately 1.5 as indicating strong bonding through silicate polymerisation. Every paste-side ratio measured here at 10–30% replacement lies well above that range (Table 4), reflecting portlandite accumulation rather than the formation of load-bearing C–S–H. Siad et al. [32] reported Ca/Si between 1.06 and 1.91 around rubber in engineered cementitious composites, with interfacial gaps averaging 8.8 µm at crumb-rubber edges and nearly 2 µm at powder-rubber margins. The widths in Table 6 exceed those sectioned values by roughly an order of magnitude, as expected for fracture-surface measurement.

Nano-indentation provides a complementary micromechanical perspective. Momotaz et al. [30] used statistical nano-indentation to measure an intrinsic interfacial transition zone thickness of 50–65 µm between tyre-derived aggregate and cement paste. When the analysis accounted for interfacial gaps and clustered porosity, the total width of the mechanically weak zone increased to 80–100 µm, substantially larger than in conventional natural-aggregate concrete. The indentation modulus was significantly lower than that of the bulk paste.

The broader ITZ literature supports interpreting this zone as the primary flaw in concrete. The ITZ is recognised as the weakest phase and the site of crack initiation [21,26]. Akçaoğlu et al. [49] demonstrated that larger inclusions result in thicker interfacial zones and accelerate ITZ microcracking under load, identifying the interfacial bond as the critical factor for tensile strength. Golewski [50] correlated the width of ITZ microcracks with the fracture parameters of fly ash concrete, while Erdem et al. [33] found that a denser, stronger ITZ preserves stiffness and promotes ductile behaviour under impact. Poon et al. [51] associated loose, porous interfacial hydrates around normal-strength recycled aggregate with reduced compressive strength. Vargas et al. [52] observed that lightweight aggregates form a dense, thin ITZ, the geometric opposite of the smooth, hydrophobic rubber examined here. Xiao et al. [53] showed numerically that the ratio of interfacial to matrix properties governs the stress–strain response of the composite, so the wide, compliant interfaces reported in Section 3.2 bear directly on the macroscopic behaviour.

### 4.3. Slump Response and Fresh-State Behavior

The two slump trends correspond to established mechanisms for rubber in fresh concrete. The low water absorption of rubber leaves excess free water in the paste, and its smooth surface reduces interparticle friction [15,19,34], which accounts for the gain at 10% fine rubber reported in Section 3.1.1. Above 20% replacement, the balance reverses: large, deformable, angular granules raise the viscous resistance of the mixture and interlock during flow [14]. Khaloo et al. [35] reported a comparable decline as tyre-aggregate concentration increased. Su et al. [48] observed higher slump with larger particles at a single 20% dosage, provided the particles were no coarser than 3 mm; the 5–20 mm granules used here, at replacement levels up to 50%, fall outside that range, and the divergence indicates that granule interlocking rather than surface character governs at higher dosages.

### 4.4. Density, Interfacial Porosity and the Durability Outlook

Benazzouk et al. [41] reached the RILEM class II lightweight requirement at 50% rubber content in a cement composite, at a rubber volume fraction similar to that of M10, which places the densities reported in Section 3.1.4 within the published range. The step in density deficit between M8 and M9 is consistent with the interfacial porosity imaged in Section 3.2.

The transport behaviour of rubberised concrete remains an unresolved issue. Benazzouk et al. [41] reported reduced sorptivity and limited water propagation as rubber content increased. Islam et al. [6] observed adequate performance of rubberised concrete under chloride exposure during wet–dry cycles. Interfacial voids, particularly those concentrated at rubber surfaces, generally serve as ingress pathways [22,24]. No exposure class should be assigned until measured absorption (NM EN 12390-8) and mercury-intrusion porosimetry have clarified the pore structure.

### 4.5. Toughness, Energy Absorption and the Mix Ranking

The ranking presented in Table 7 draws on a property conferred by rubber rather than one it diminishes. Atahan and Yücel [8] observed a reduction in peak impact load and an enhancement in energy dissipation as rubber content increased, identifying high-rubber mixtures as appropriate for roadside safety structures. Du et al. [47] found that the dynamic increase factor of rubberised concrete increases with higher rubber content, with optimal energy absorption achieved at 30% rubber across three strain rates. Benazzouk et al. [41] reported a significant increase in strain capacity and toughness, despite reductions in strength.

Hybridisation operates under a similar rationale. Fu et al. [18] and Xiong et al. [17] observed the restoration of mechanical performance when steel or glass fibres were incorporated into the rubberised matrix. Ayhan et al. [54] reported 92.9% strength retention at 400 °C for a rubber–steel-fibre hybrid, against 72.2% for plain concrete, indicating that the energy-absorbing application extends to fire scenarios. In situ testing of ordinary concrete at elevated temperature shows how far mechanical and microstructural deterioration proceed under thermal exposure [55], which is the behaviour any rubberised mix intended for such service would have to be measured against, and which mechanical characterisation at ambient temperature cannot establish. M9 and M10, which exhibit compressive strength reductions of 70.0% and 81.8% respectively, are placed in this category. Impact and energy-absorption properties were not measured in the present programme, and the placement is inferred from the cited literature rather than demonstrated here.

### 4.6. Routes to Interface Improvement

Interfacial challenges in rubberised concrete are both chemical and geometric. Surface pre-treatments modify the substrate to which the cement paste bonds. Pham et al. [22] reported significant enhancement of the rubber–matrix bond and improved transfer properties by applying a styrene-butadiene copolymer coating. Kashani et al. [56] demonstrated that a silica fume coating serves as a nucleation site for additional C–S–H formation, narrowing the interfacial gap and improving adhesion. Agrawal et al. [20] achieved a compressive strength of 61.5 MPa by treating rubber fibres with a combination of NaOH and silica fume, resulting in performance nearly equivalent to that of the reference mix at 10% replacement. On the binder side, calcium-consuming additives directly target the portlandite shells that create weak interfacial zones [40,50].

## 5. Conclusions

Eleven concrete mixes incorporating fine (2.5–5 mm) and coarse (5–20 mm) waste tyre rubber at 10–50% volume replacement were evaluated in accordance with NM EN standards. The rubber–paste interface in six mixes was characterised using scanning electron microscopy and energy-dispersive X-ray spectroscopy (SEM/EDX). The principal findings are as follows:

• Workability exhibited two size-dependent trends. Fine rubber increased the slump to 114 mm at 10% replacement but reduced it to 55 mm at 50%. Coarse rubber maintained the reference slump at 10% replacement and reduced it to 30 mm at 50% through granule interlocking.
• Strength decreased monotonically with increasing rubber content and was influenced by particle size. At 50% replacement, compressive strength losses reached 66.3% for fine rubber and 81.8% for coarse rubber. Coarse rubber consistently produced greater reductions at all levels (−31.0% versus −8.5% at 10% replacement).
• Particle size, not dosage alone, governs the loss. At an equal superplasticiser dosage, each coarse-rubber mix was weaker than its fine-rubber counterpart despite containing 10 vol. % less rubber. Flexural strength was retained better than compressive strength at intermediate and high fine-rubber contents, and the two losses were comparable throughout the coarse-rubber series.
• Density decreased by 8.0% for fine rubber and 17.2% for coarse rubber at 50% replacement. Mixes M9 and M10 fell below the EN 206 lightweight concrete threshold. The 189 kg/m^3^ density reduction between M8 and M9 exceeded the substituted mineral mass by 124 kg/m^3^. Under saturated surface-dry weighing, this deficit is a lower bound on void content, and compaction failure at high coarse-rubber dosage is the interpretation most consistent with a step change confined to the coarse series. Direct porosity measurement is required to confirm it.
• Interfacial chemistry analysis identified the ITZ as the primary site of degradation. All rubberised mixes exhibited increased paste-side calcium enrichment relative to the reference mix (Ca/Si = 1.24), with a maximum of 8.09 for the 10% coarse-rubber mix. The presence of a sulphur–zinc signature confirmed the presence of a vulcanised interface. The wide range of Ca/Si values at 50% replacement (1.52 to 10.78 within M5) shows that a single analysed field is not representative of interfacial chemistry at high dosage and does not indicate improved bonding. Whether the spread reflects matrix heterogeneity or sampling from non-representative domains cannot be resolved by the present data.
• The apparent debonding-zone width increased monotonically from 10–25 µm at 10% fine rubber to 100–200 µm at 50% coarse rubber. At 10% replacement, the coarse-rubber value was approximately three times the fine-rubber value, and about twice at 50%, supporting flaw-size scaling as the origin of the particle-size effect. These widths are measured on fracture surfaces and used for comparison between mixes; verification of intrinsic interfacial thickness requires backscattered-electron imaging of polished sections.
• Porosity evolved from isolated pores at low rubber dosages to interconnected interfacial void networks at 50% replacement, assessed qualitatively. Transport-property and durability testing should be prioritised in future research.

On mechanical grounds, 10% fine rubber provides the most favourable semi-structural balance. Intermediate dosages rank next, while 40–50% mixes fall into the energy-absorbing category. These rankings are preliminary and subject to durability verification for any intended exposure. The results support a graded valorisation strategy for Moroccan end-of-life tyres, matching dosage and particle size to the intended application rather than seeking a single optimum.

The limitations identified above define the priorities for further work:

• Interface verification and treatment: backscattered-electron imaging of polished, carbon-coated sections to establish the intrinsic gap width and porosity gradient across the ITZ, together with elemental area mapping at high magnification; and systematic study of rubber pre-treatment (alkali wash, silane coupling, plasma treatment, cement-slurry coating) and its effect on the interfacial Ca/Si profile, the debonding-zone width and strength.
• Porosity and transport: fresh air content by the pressure method (NM EN 12350-7), water absorption to NM EN 12390-8, mercury-intrusion porosimetry and nuclear magnetic resonance relaxometry, which resolves pore-structure evolution in cemented composites [46], to confirm the void content inferred in Section 3.1.4; and durability testing covering chloride ingress, freeze–thaw, carbonation and acid or sulphate resistance to the relevant NM EN standards.
• Supplementary cementitious materials: combining rubber with fly ash, silica fume or slag to shift the interfacial chemistry toward C–S–H.
• Long-term behaviour: creep, shrinkage, and re-examination of the interface at later ages to quantify gap closure under continued hydration, particularly for M1 and M6.
• Single-variable verification: a dedicated series cast at fixed superplasticiser dosage to isolate the absolute magnitude of the rubber penalty within each series.

## Figures and Tables

**Figure 1:**
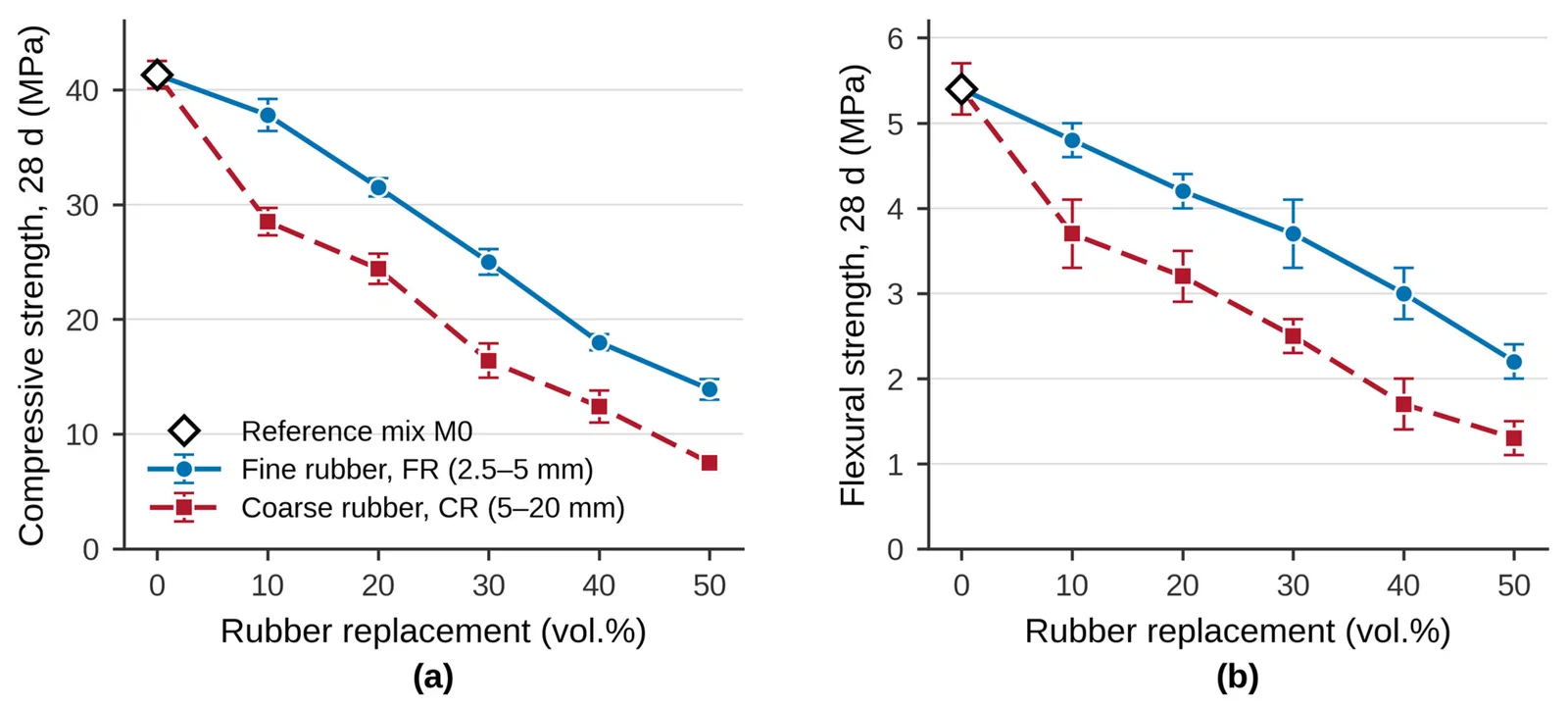
Compressive and flexural strength against rubber replacement for the fine-rubber (FR) and coarse-rubber (CR) series: (**a**) compressive strength at 28 days; (**b**) flexural strength at 28 days. Error bars show ± one standard deviation (*n* = 3).

**Figure 2:**
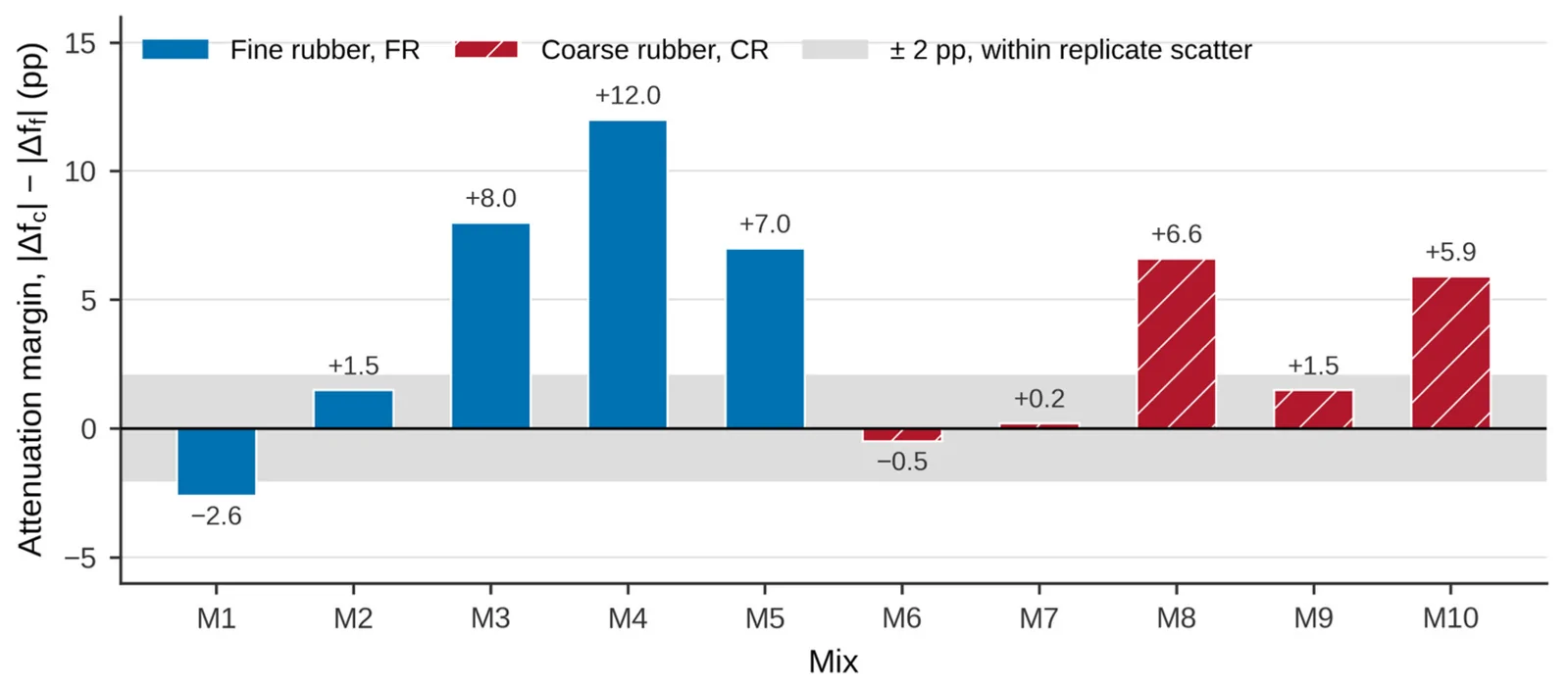
Attenuation margin, |Δ*f*_c_| − |Δ*f*_t_|, for each mix in percentage points. Positive values indicate that flexural strength was retained better than compressive strength.

**Figure 3:**
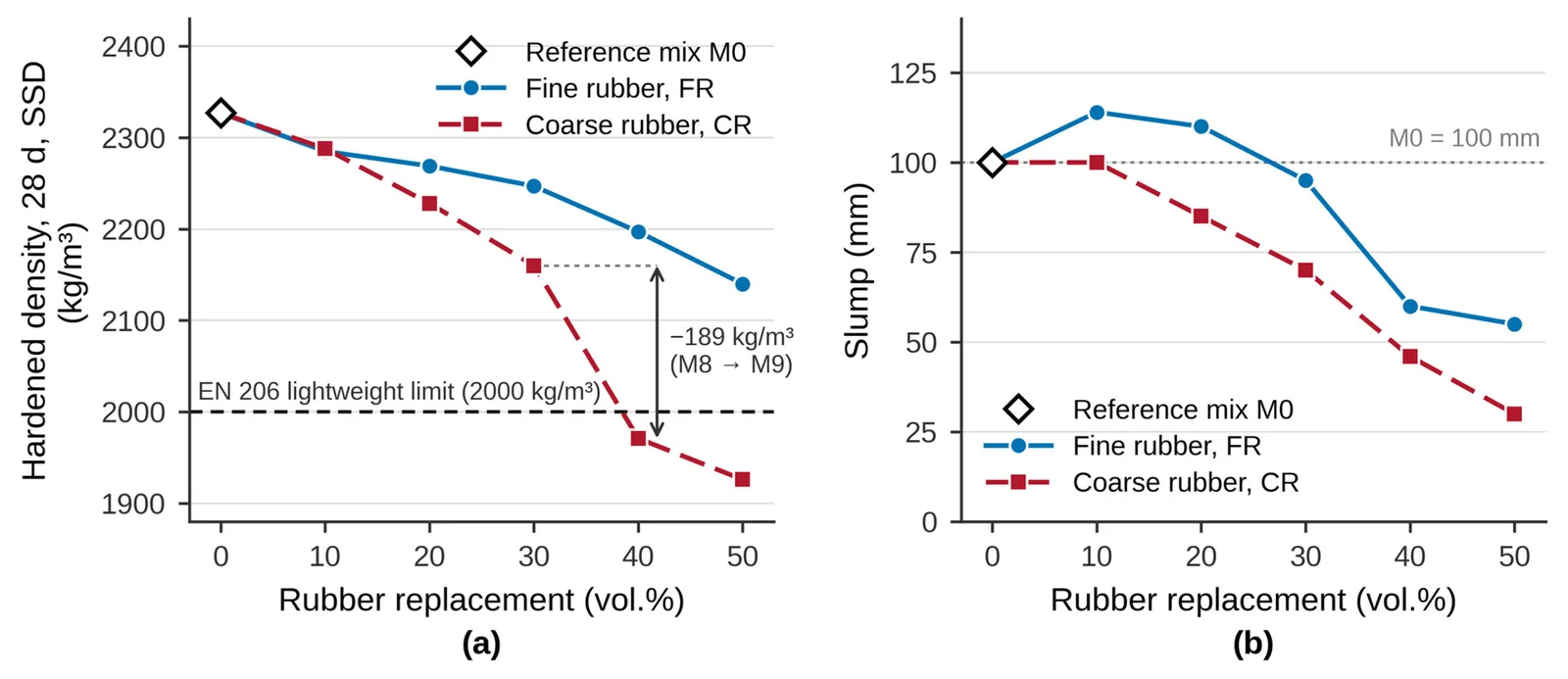
(**a**) Measured 28-day hardened density and (**b**) slump against rubber replacement. The dotted line in (**a**) marks the EN 206 lightweight boundary (2000 kg/m^3^), crossed by M9 and M10.

**Figure 4:**
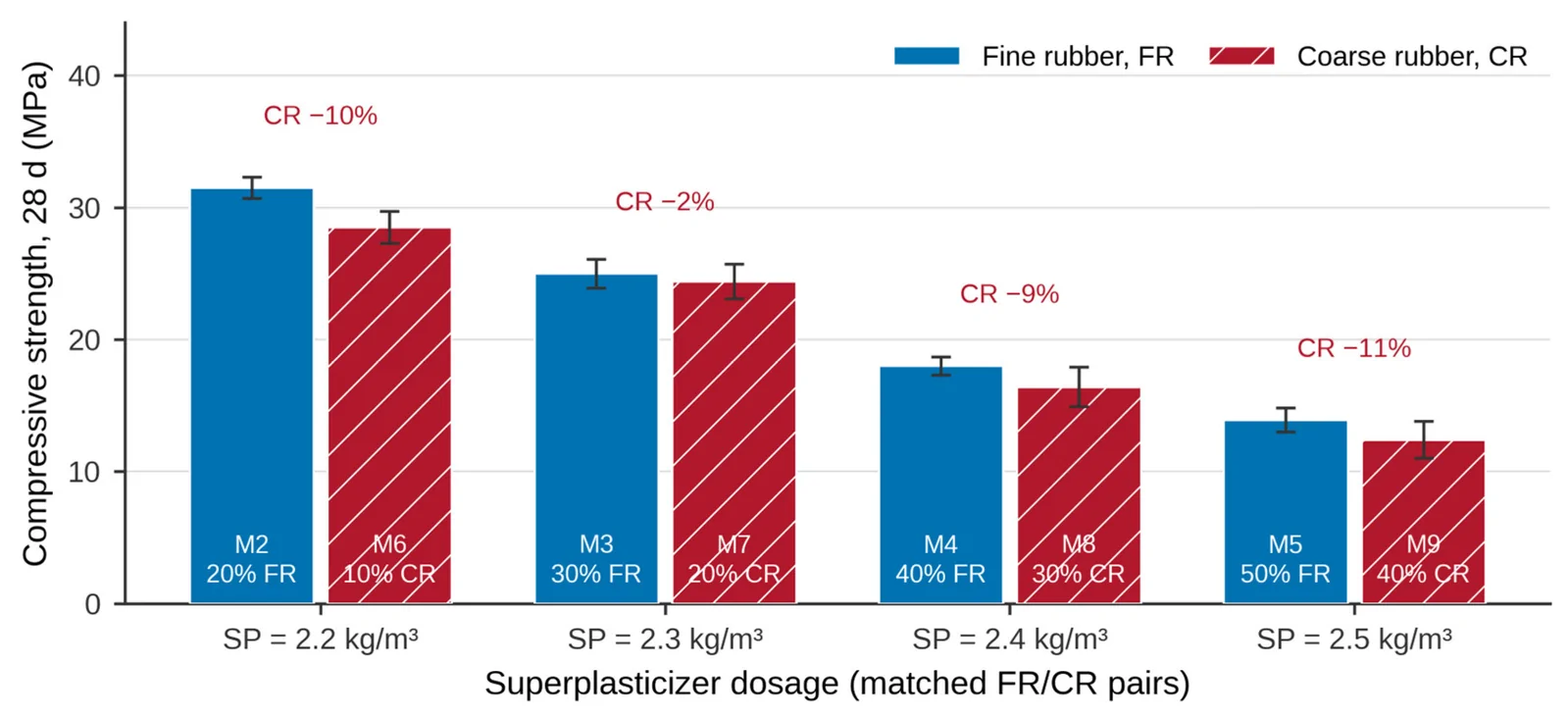
Compressive strength at 28 days for the four fine-rubber and coarse-rubber pairs cast at identical superplasticiser dosage.

**Figure 5:**
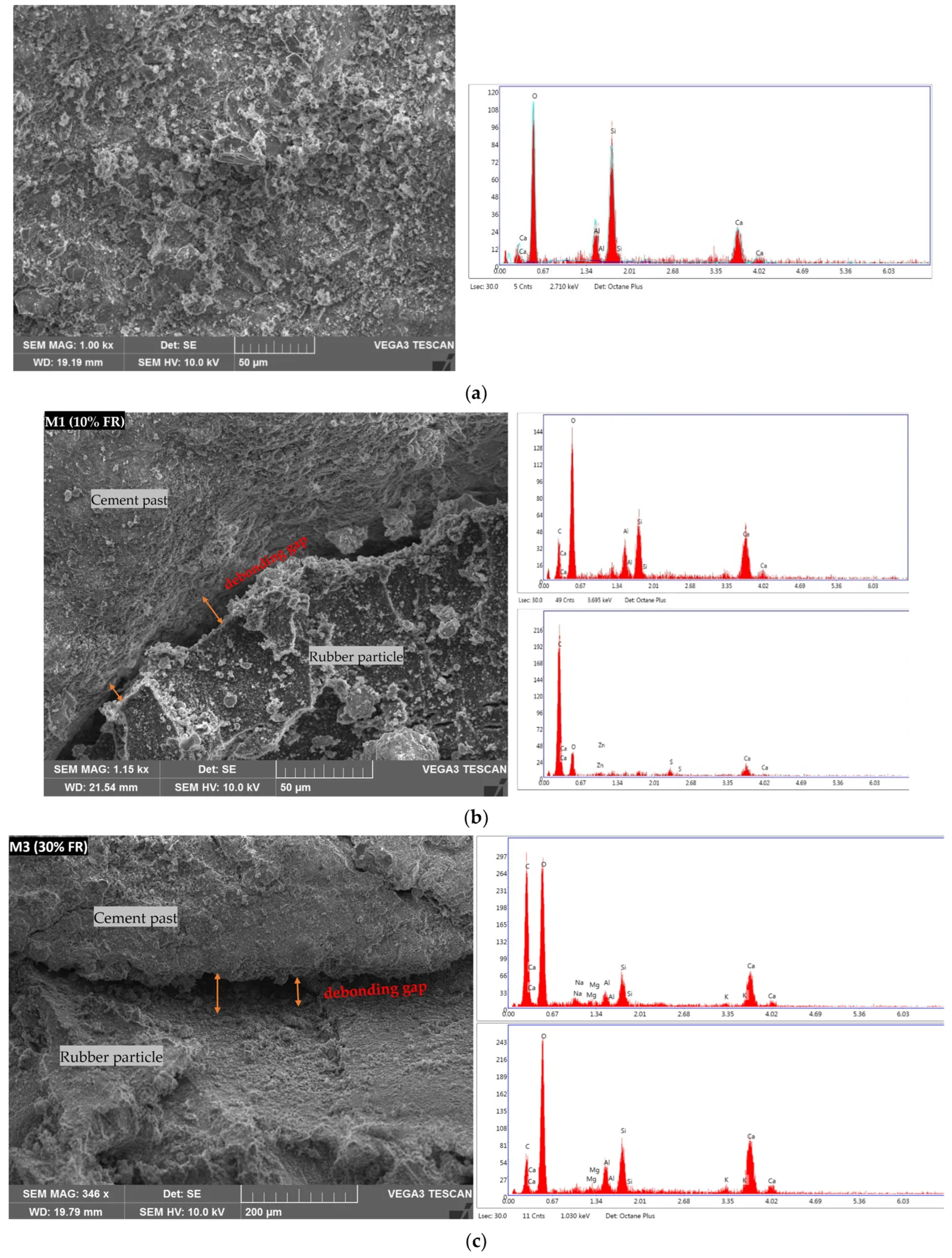

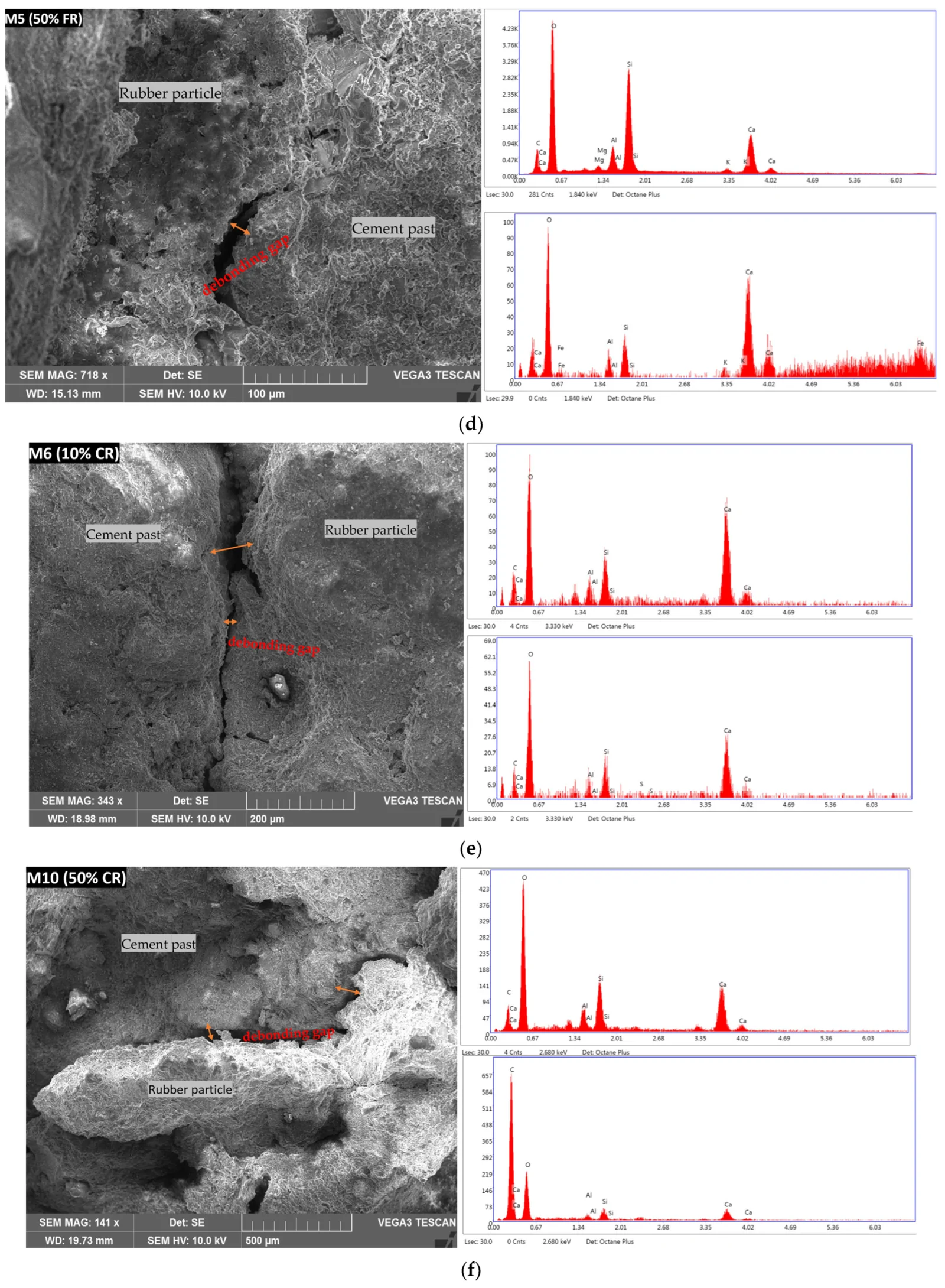
(**a**). SE micrograph of the M0 bulk cement paste and the corresponding EDX spectrum of the analysed area (bottom), showing the dense, homogeneous hydrated matrix and the Ca–Si-dominated composition (Ca/Si = 1.24). (**b**). SE micrograph of M1 (10% FR) showing a rubber particle embedded in the cement matrix, with the fracture-surface debonding gap marked, and the EDX spectrum of the paste-side field. (**c**). SE micrograph of M3 (30% FR) showing the widened debonding gap and incipient pore clusters at the rubber surface, with the EDX spectrum of the boundary-overlapping field. (**d**). SE micrograph of M5 (50% FR) showing the discontinuous paste matrix and rubber network, with the EDX spectrum of the silicon-rich paste-side field. (**e**). SE micrograph of M6 (10% CR) showing the wide debonding gap around a coarse-rubber granule, with the EDX spectrum of the paste-side field. (**f**). SE micrograph of M10 (50% CR) showing the friable, discontinuous paste network, wide debonding gaps and unloaded-state microcracking, with the EDX spectrum of the paste-side field.

**Figure 6:**
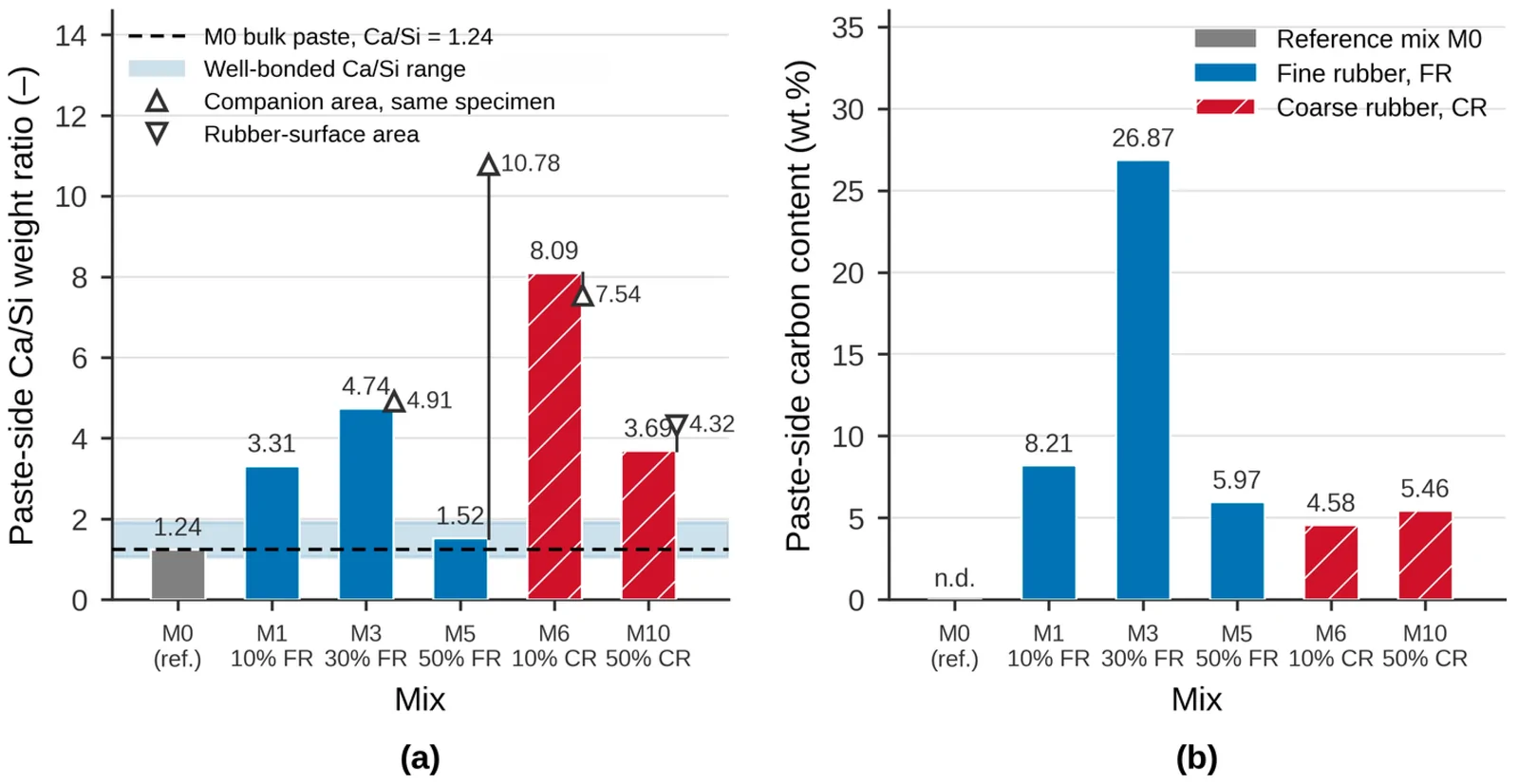
EDX-derived interfacial indicators: (**a**) paste-side Ca/Si ratio, with the M0 bulk-paste value shown as a reference line; (**b**) paste-side carbon content.

**Figure 7:**
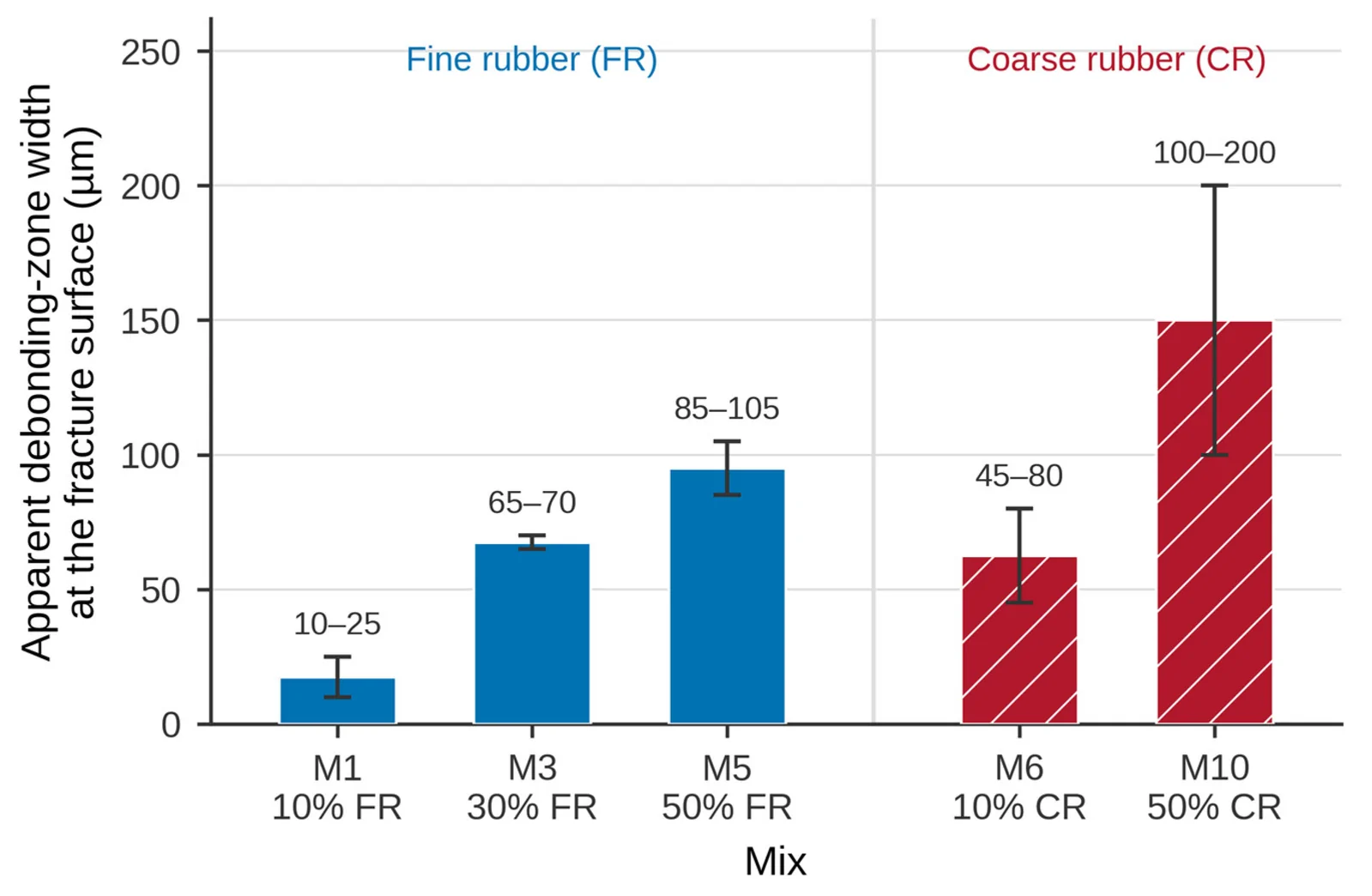
Apparent debonding-zone width at the fracture surface against mix; bars show the mean of three readings and whiskers the measured range. Fine-rubber mixes in blue, coarse-rubber in red.

**Table 1:** Aggregate and rubber properties.

Property	Sand 0/4	Limestone 4/10	Limestone 10/20	Fine Rubber	Coarse Rubber	Standard
Size (mm)	0–4	4–10	10–20	2.5–5	5–20	NM EN 12620
Particle density (Mg/m^3^)	2.632	2.625	2.629	1.15	1.15	NM EN 1097-6
Water absorption (%)	0.44–0.51	0.23–0.32	0.23–0.32	negligible	negligible	NM EN 1097-6
Los Angeles coefficient (%)	—	20	18	—	—	NM EN 1097-2
Micro-Deval coefficient (%)	—	12	10	—	—	NM EN 1097-1
Flakiness index (%)	—	15	10	—	—	NM EN 933-3
Sand equivalent (%)	65	—	—	—	—	NM EN 933-8
Methylene blue value	0.60	—	—	—	—	NM EN 933-9

**Table 2:** Mix proportions (kg/m^3^ of concrete). Cement, water, and w/c are constant; only the replaced aggregate, the rubber, and the superplasticiser (SP) vary.

Mix	Replacement by Volume%	Cement	Water	W/C	Sand	Coarse	Rubber	SP
M0	0%	400	180	0.45	631.3	1148.4	0	2.0
M1	10% FR	400	180	0.45	568.2	1148.4	27.6	2.1
M2	20% FR	400	180	0.45	505.0	1148.4	55.2	2.2
M3	30% FR	400	180	0.45	441.9	1148.4	82.8	2.3
M4	40% FR	400	180	0.45	378.8	1148.4	110.3	2.4
M5	50% FR	400	180	0.45	315.7	1148.4	137.9	2.5
M6	10% CR	400	180	0.45	631.3	1033.6	50.3	2.2
M7	20% CR	400	180	0.45	631.3	918.7	100.5	2.3
M8	30% CR	400	180	0.45	631.3	803.9	150.8	2.4
M9	40% CR	400	180	0.45	631.3	689.0	201.0	2.5
M10	50% CR	400	180	0.45	631.3	574.2	251.3	2.6

**Table 3:** Mechanical and physical properties. Strength values represent means ± one standard deviation from three replicates. Hardened densities were measured in accordance with NM EN 12390-7 under saturated surface-dry conditions.

Mix	Repl. Vol. %	Slump (mm)	*f*_c_ 28 d (MPa)	Δ*f*_c_	*f*_t_ 28 d (MPa)	Δ*f*_t_	Hard. ρ 28 d (kg/m^3^)	Δρ	Δ*f*_t_/Δ*f*_c_
M0	0%	100	41.3 ± 1.2	–	5.4 ± 0.3	–	2327	–	–
M1	10% FR	114	37.8 ± 1.4	−8.5%	4.8 ± 0.2	−11.1%	2285	−1.8%	1.31
M2	20% FR	110	31.5 ± 0.8	−23.7%	4.2 ± 0.2	−22.2%	2269	−2.5%	0.94
M3	30% FR	95	25.0 ± 1.1	−39.5%	3.7 ± 0.4	−31.5%	2247	−3.4%	0.80
M4	40% FR	60	18.0 ± 0.7	−56.4%	3.0 ± 0.3	−44.4%	2197	−5.6%	0.79
M5	50% FR	55	13.9 ± 0.9	−66.3%	2.2 ± 0.2	−59.3%	2140	−8.0%	0.89
M6	10% CR	100	28.5 ± 1.2	−31.0%	3.7 ± 0.4	−31.5%	2288	−1.7%	1.02
M7	20% CR	85	24.4 ± 1.3	−40.9%	3.2 ± 0.3	−40.7%	2228	−4.3%	1.00
M8	30% CR	70	16.4 ± 1.5	−60.3%	2.5 ± 0.2	−53.7%	2160	−7.2%	0.89
M9	40% CR	46	12.4 ± 1.4	−70.0%	1.7 ± 0.3	−68.5%	1971	−15.3%	0.98
M10	50% CR	30	7.5 ± 0.8	−81.8%	1.3 ± 0.2	−75.9%	1926	−17.2%	0.93

**Table 7:** Preliminary mix ranking by 28-day mechanical performance. Densities are the measured 28-day hardened values in Table 3. No entry constitutes a service recommendation.

Mix	*f*_c_ 28 (MPa)	ρ (kg/m^3^)	Slump (mm)	Mechanical Class	Candidate Uses, Subject to Durability Verification
M0	41.3	2327	100	C30/37	Structural reference
M1	37.8	2285	114	C25/30	Impact-loaded pavements, industrial flooring, lightly loaded walls [40]
M2–M3	25.0–31.5	2247–2269	95–110	C20/25	Partition walls, acoustic blocks, precast elements [1,13]
M4–M5	13.9–18.0	2140–2197	55–60	Non-structural	Sound barriers, pedestrian surfaces, sports flooring [13,43]
M6	28.5	2288	100	C20/25 *	Non-critical sub-structures and drainage in non-aggressive environments [6]
M7–M8	16.4–24.4	2160–2228	70–85	Lightweight fill	Road sub-base, embankment fill [36]
M9–M10	7.5–12.4	1926–1971	30–46	Energy absorbing	Crash barriers, protective structures, playground surfacing, vibration isolation [8,30,47]

* Although M6 meets the compressive-strength requirement for class C20/25, its interface exhibits the greatest degradation among the mixes. The mix has the highest paste-side Ca/Si ratio measured in the study (8.09) and a debonding-zone width of 45–80 µm, approximately three times that of M1 at the same replacement level (Section 3.2.6 and Section 3.2.8). These interfacial characteristics indicate that compressive-strength classification alone may not fully reflect its expected in-service performance; M6 is therefore flagged accordingly.

## Data Availability

The original contributions presented in this study are included in the article. Further inquiries can be directed to the corresponding author.

## Abbreviations

The following abbreviations are used in this manuscript:

BSE	Backscattered electron
Ca/Si	Calcium-to-silicon weight ratio
CEM I	Portland cement, type I (NM EN 197-1)
CH	Calcium hydroxide (portlandite)
CR	Coarse rubber
C–S–H	Calcium silicate hydrate
EDX	Energy-dispersive X-ray spectroscopy
EN	European Standard (Norme Européenne)
eZAF	Enhanced ZAF matrix-correction routine
FR	Fine rubber
ITZ	Interfacial transition zone
MDE	Micro-Deval coefficient, wet method
NM	Moroccan Standard (Norme Marocaine)
QBSE	Quantitative backscattered electron imaging
RC	Rubberised concrete
RILEM	International Union of Laboratories and Experts in Construction Materials, Systems and Structures
SEM	Scanning electron microscopy
SP	Superplasticizer
SSD	Saturated surface-dry
TDA	Tyre-derived aggregate
W/C	Water-to-cement ratio
ZAF	Atomic number, absorption and fluorescence correction
